# Exploring Therapists’ Experiences of an Educational Website to Support Telehealth Delivery of Constraint-Induced Movement Therapy

**DOI:** 10.3390/healthcare13020159

**Published:** 2025-01-15

**Authors:** Kate Makroglou, Nicola Fearn, Bianca Portelli, Helen Badge, Jessamy Boydell, Anna Kilkenny, Annie Meharg, Lauren J. Christie

**Affiliations:** 1 Allied Health Research Unit, St Vincent’s Health Network Sydney, Darlinghurst, NSW 2010, Australialauren.christie@svha.org.au (L.J.C.); 2 School of Allied Health, Faculty of Health Sciences, Australian Catholic University, North Sydney, NSW 2060, Australia; 3 Arm’s Reach Occupational Therapy, Bristol BS7 9HW, UK; info@armsreach-ot.co.uk; 4 Align Health, Cambridge 3434, New Zealand; 5 Centre for Health and Social Practice, Wintec, Te Pūkenga, Hamilton 3240, New Zealand; 6 Independent Researcher, Bath BA1 5SN, UK

**Keywords:** distance education, occupational therapy, physical therapy, telerehabilitation

## Abstract

**Purpose:** Constraint-induced movement therapy (CIMT) is an evidence-based intervention for arm recovery after acquired brain injury. Clinician knowledge, time and confidence in delivering CIMT are established barriers to the routine use of CIMT in practice. CIMT delivery via telehealth is one option to help overcome these barriers. This study aimed to understand clinician experiences of using an educational website and if the education and online resources contributed to their self-reported use of constraint-induced movement therapy via telehealth (TeleCIMT) in practice. **Materials and Methods:** Data were collected from a purposive sample of therapists registered to use the TeleCIMT website and website analytics. An online survey explored participants’ experience with CIMT delivery (both face to face and via telehealth), their perceptions of the website, and barriers and enablers to TeleCIMT implementation using the Capability, Opportunity, Motivation—Behaviour model. Website analytics were used to evaluate website traffic and resource use. Data were analysed using descriptive statistics (quantitative data) and content analysis (qualitative data). **Results:** Forty therapists responded to the survey; 72.5% (n = 29) of the respondents were occupational therapists, and 37.5% (n = 15) had delivered TeleCIMT. Most of the participants agreed that the website was easy to navigate (n = 26, 90%) and felt that they had the knowledge (n = 28, 96.6%) and skills (n = 24, 82.7%) to deliver TeleCIMT. The enablers to TeleCIMT included motivation to implement learnings from the website, confidence in delivering the programme, and the convenience of remote delivery. The perceived barriers to TeleCIMT use included limited access to technology and the availability of a client supporter to enable engagement in TeleCIMT. The resources used most frequently by the respondents were the participant preparation pack and participant programme pack. Shorter video learning modules (<11 min in duration) had greater engagement than longer video learning modules. **Conclusions:** Whilst online education and resources may enhance clinician knowledge of constraint-induced movement therapy and telehealth delivery, other barriers such as lack of technology access, may need to be addressed through additional learning and implementation strategies to support the routine use of TeleCIMT in practice.

## 1. Introduction

Constraint-induced movement therapy (CIMT) is an intensive, evidence-based intervention that improves motor function of the affected arm and increases engagement in meaningful, everyday occupations after acquired brain injury [1,2]. CIMT comprises three essential components: (i) wearing a mitt or restraint on the unaffected hand for at least six hours per day, for at least two weeks (ii) completing repetitive, task-oriented training of the affected arm for at least two hours per day and (iii) a transfer package to support programme adherence and the generalisation of skills into daily life [2].

While CIMT is a strongly recommended intervention for arm recovery after stroke in multiple national clinical guidelines [3,4], the barriers to CIMT implementation in practice have been well established. These include therapist’s lack of time, knowledge and skills, transport for clients to attend daily appointments and environmental considerations such as the suitability of therapy spaces [5,6]. To address some of these barriers, alternative modes of CIMT delivery have been explored, including CIMT via telehealth (TeleCIMT) [7,8,9,10]. TeleCIMT is a three-week CIMT programme, delivered in a semi-supervised format while still maintaining the three essential components mentioned above [10]. A TeleCIMT programme has less direct therapist input than is typical for a face-to-face programme, with clients receiving three one-hour intensive therapy sessions per week via video calls, and two brief, fifteen to thirty-minute telephone or video calls two days per week to monitor practice and progress activities. Outside of structured therapy times, clients follow a pre-set programme using a TeleCIMT workbook with or without the support of a carer [10].

Despite its potential to overcome the barriers to CIMT delivery, TeleCIMT is not routinely offered by clinicians, possibly due to a lack of education, training and resources to support therapists to deliver telehealth interventions. In a rehabilitation context, online learning has been demonstrated to be effective in increasing clinician knowledge in specialist, evidence-based neurorehabilitation interventions [11,12].

TeleCIMT.com was created in 2020, providing clinicians with free education, training and resources to support TeleCIMT implementation in practice. The website comprises three sections: (i) therapist learning resources, (ii) therapist programme resources and (iii) TeleCIMT participant and supporter resources including programme brochures and therapy packs, as well as supporting videos.

To date, clinicians’ perceptions of whether the TeleCIMT.com website has supported them to implement TeleCIMT more routinely in practice have not been investigated. We aimed to understand clinician experiences of using the website and if the education and resources contributed to their self-reported use of TeleCIMT in practice. The specific research questions were:

1. What is the level of user engagement with the TeleCIMT learning modules and resources?
2. What are users’ experiences and opinions regarding the ease of use and design of the TeleCIMT website?
3. What are the clinician-reported barriers and enablers to the implementation of TeleCIMT in practice?

## 2. Methods

A mixed methods evaluation was undertaken, using a cross-sectional online survey design and website analytic data.

### 2.1. Eligibility

Website users were eligible to participate in the survey if they were occupational therapists, physiotherapists, students of these disciplines or allied health assistants; had registered to use the TeleCIMT website using a valid email address and had accessed the website between December 2020 and December 2023.

### 2.2. Recruitment and Consent

Purposive sampling was used to recruit clinician survey respondents via email using website registration data. A reminder email invitation was sent up to four times to those who had not accessed the survey or had partially completed it. No incentives to complete the survey were offered. An electronic participant information sheet was embedded at the start of the survey, and informed consent was implied through survey completion.

### 2.3. Data Collection

The online survey was developed in REDCap [13,14,15]. The survey included both closed and open-ended questions and comprised four sections: (i) demographics, (ii) experiences with CIMT delivery, (iii) website use and design; and (iv) self-reported barriers and enablers to implementing TeleCIMT. The questions regarding barriers and enablers were informed by the Capability, Opportunity, Motivation-Behaviour (COM-B) model [16]. A copy of the survey questions is available from the authors upon request. Website traffic data were collected from December 2020 to December 2023.

### 2.4. Data Analysis

De-identified survey and website analytics data were imported into Statistical Package for Social Sciences (SPSS) version 28.0.1.0 [17] and analysed using descriptive statistics. Qualitative data were analysed using content analysis.

## 3. Results

### 3.1. Demographics

There were 667 potentially eligible respondents who were invited to complete the survey; 45 (6.7%) responded. Data from five respondents were excluded from the analysis and there was a final sample size of 40 respondents (6%). The respondent demographics are described in Table 1.

### 3.2. Experience in Delivering CIMT

Most of the respondents had delivered CIMT face-to-face (n = 27, 67.5%) and had delivered an average of five face-to-face programmes in the past two years (median = 2, IQR 0.0–5.0). Most of the respondents reported that they had tried to use CIMT in their practice on occasion (n = 22, 55%), ten respondents (25%) regularly used CIMT in their practice, and eight respondents (20%) had never used it. A lower proportion of respondents had delivered CIMT via telehealth (n = 15, 37.5%), primarily to people with stroke (n = 14, 35%). The perceived benefits of TeleCIMT included increased use and function of the affected arm, as well as the flexibility of remote delivery. The respondents described the following:

*“…improved functional use of their affected upper limb...convenience of being able to do therapy in their own home”*.

(Respondent 190)

*“All achieved overall functional improvements and goals…”*.

(Respondent 209)

### 3.3. Website Engagement

Website analytics demonstrated that the videos with the highest number of views were “Introduction to CIMT” (1868 views), “Structured Training in CIMT: Shaping” (1291 views) and “Mitt wear in CIMT” (913 views). Modules that were less than 11 min in duration were watched for 71.5% of their total time, whilst longer videos were generally only engaged with for around half of their total duration.

The therapist learning modules were accessed by 87.5% (n = 35) of respondents, who reported that all the modules had improved their knowledge of CIMT. Most of the respondents had also accessed and used the therapist resources (n = 32, 80%). The respondents found the shaping and task practice libraries to be the most helpful resources for supporting their practice. The respondents had provided clients with the TeleCIMT participant preparation pack (n = 16, 40%) and TeleCIMT participant programme packs (n = 16, 40%) most often, usually via a printed copy.

Most survey respondents agreed that the website was easy to navigate (n = 28, 70%) and that they would recommend the website to others (n = 33, 82.5%). Suggestions for improvement included expanding on the task practice and shaping practice libraries, condensing the length of the programme pack and updating the video resources to include stroke survivors with lived experience of completing CIMT.

### 3.4. Clinician-Perceived Barriers and Enablers to Implementing TeleCIMT in Practice

**Figure 1:**
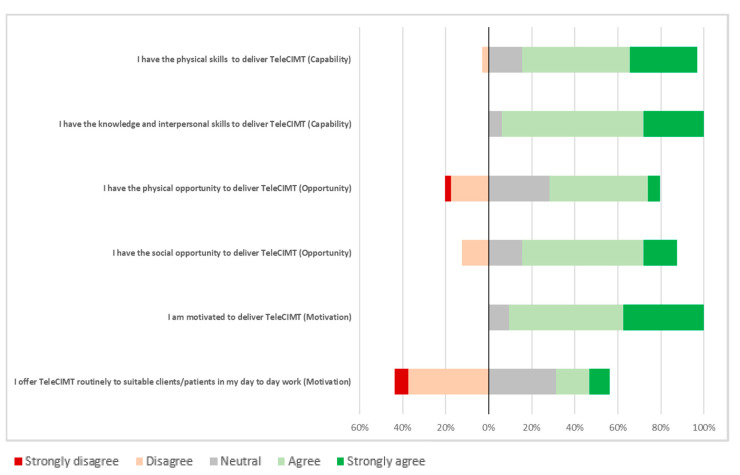
Barriers and enablers to TeleCIMT in practice using the COM-B model of behaviour.

Thirty-two respondents rated their agreement with statements about the barriers to and enablers of TeleCIMT implementation in practice (Figure 1). The majority perceived that they had the capability to provide TeleCIMT, responding that they felt that they had the necessary knowledge (strongly agree or agree n = 30, 93.7%) and skills (strongly agree or agree n = 26, 81.3%). The respondents also reported sufficient social opportunity, with adequate support from their colleagues available to deliver TeleCIMT (strongly agree or agree n = 23, 71.9%). Most were motivated to implement TeleCIMT, agreeing that they had a desire to deliver TeleCIMT to clients (strongly agree or agree n = 29, 90.6%). Despite these enablers, more than half (n = 24, 60%) of the respondents stated that they did not routinely consider TeleCIMT when planning interventions (neutral, disagree or strongly disagree n = 24, 75.1%). Only around half of the respondents felt that they had adequate time and materials to deliver TeleCIMT (n = 17, 53.1% agreed or strongly agreed).

For some of the respondents, the website and resources provided a way to overcome the challenges related to the time commitment needed to run a programme. One respondent stated the following:

*“I think a barrier to using CIMT in everyday practice can be the time needed to set up the programme and deliver it, especially in the public sector. This website really assists in reducing the amount of time needed for prep, and hopefully that can drive change in our workplace”*.

(Respondent 504)

## 4. Discussion

This study evaluated TeleCIMT.com, which aims to support therapists to implement a telerehabilitation programme using constraint-induced movement therapy. The respondents found the website easy to navigate, reported that the training modules improved their knowledge of CIMT and that they are using the freely available resources; however, the proportion of those who routinely considered delivering this intervention in practice remained relatively low. This indicates that barriers consistent with the broader literature on CIMT implementation remained [5,18,19,20]. The enablers to implementing TeleCIMT were therapist motivation and an increase in self-reported knowledge and skills following website use.

The feedback on the website was largely positive, contributing to the growing body of evidence for the use of online learning to teach health professionals complex interventions [12,21]; however, self-directed learning alone is insufficient to support practice change [22,23]. The facilitation of ongoing skill development and peer learning may increase TeleCIMT uptake [24]. Opportunities for interaction with an online learning community to share ideas and provide feedback may also be useful [25,26,27]. For example, communities of practice facilitate the exchange of knowledge, skills and best practices [28,29] and have previously been used to support CIMT implementation [19].

Website analytics indicated that there was low engagement with longer training videos. One factor could be the didactic nature of the pre-recorded videos. Promoting collaboration between learners and educators has been shown to be crucial for user engagement in online learning environments [25,30,31]. Including elements such as self-directed learning, embedded feedback and peer support into the course design could improve user engagement [32,33].

Suggestions for website improvements like streamlining resources and providing more therapy activities in the shaping and task practice libraries to help with implementation will be considered in future website updates.

## 5. Limitations

The survey had a low response rate (6%) and a small sample size (40) considering the wide distribution of the survey internationally. One possible reason for this low response rate may have been the strict inclusion criteria, requiring the respondents to have used the TeleCIMT resources in practice in order to be eligible. Many potential respondents may have had an initial interest in the resources and registered on the website, but not had an opportunity to use the resources in practice. Another possible reason is that the potential respondents may have felt that the materials were not useful for their practice.

Another study limitation was that the data collected relied on respondents’ self-report, and no objective measures of CIMT implementation such as observations, file audits or measurement of change in self-reported knowledge and skills before and after using the resources were completed. Finally, the time between the respondents accessing the website and completing the survey also varied and was not accounted for in the analysis.

## 6. Conclusions

This study provides preliminary evidence that online education may be a helpful tool for increasing clinician knowledge of CIMT and telerehabilitation, providing an alternative way to address one of the common clinician-reported barriers to CIMT implementation—lack of knowledge. While online education may be a helpful adjunct to clinician skill development, additional support strategies are needed to overcome the barriers to TeleCIMT delivery in practice. Active implementation strategies, such as the establishment of communities of practice, and audit and feedback need to be considered and executed alongside online education to support the translation of TeleCIMT knowledge and skills into practice.

To gain a comprehensive understanding of the impact of online education on the implementation of interventions such as CIMT, future studies should consider the triangulation of self-reported implementation data with audit and observational data before and after online learning. 

## Figures and Tables

**Table 1:** Survey respondent demographics.

Characteristic	Description	n (%)
Gender	Female Male	38 (95) 2 (5)
Role	Qualified clinician	40 (100)
	Allied health student	0 (0)
Discipline	Occupational Therapy	29 (72.5)
	Physiotherapy	11 (27.5)
Years of experience in	0–5 years	16 (40)
neurological rehabilitation	6–15 years	8 (20)
	More than 15 years	16 (40)
Area/s of practice *	Acute care	5 (12.5)
	Inpatient rehabilitation	13 (32.5)
	Outpatient rehabilitation	19 (47.5)
	Community	17 (42.5)
	Other	1 (2.8)
Type of service	Public	35 (87.5)
	Private	4 (10)
	Not-for-profit organisation	1 (2.5)
Country of practice	Australia	26 (65)
	New Zealand	4 (10)
	United Kingdom	9 (22.5)
	United States of America	1 (2.5)

* The respondents may have worked in more than one practice area; therefore, proportions do not equal 100%.

## Data Availability

The data presented in this study are available upon reasonable request from the corresponding author.
